# Complete mitochondrial genome of *Penicillidia jenynsii* (Diptera: Hippoboscoidea: Nycteribiidae) and phylogenetic relationship

**DOI:** 10.1017/S003118202300032X

**Published:** 2023-06

**Authors:** Jinting Yang, Xiaobin Huang, Yujuan Wang, Huijuan Yang, Xianzheng Zhang, Xiaoyan Zheng

**Affiliations:** 1 Institute of Pathogens and Vectors, Yunnan Provincial Key Laboratory for Zoonosis Control and Prevention, Dali University, Dali, Yunnan, China; 2 Jilin Provincial Key Laboratory of Animal Resource Conservation and Utilization, Northeast Normal University, Changchun, China

**Keywords:** Bat fly, mitochondrial genome, Nycteribiidae, *Penicillidia jenynsii*

## Abstract

In recent years, bat-associated pathogens, such as 2019 novel coronavirus, have been ravaging the world, and ectoparasites of bats have received increasing attention. *Penicillidia jenynsii* is a member of the family Nycteribiidae which is a group of specialized ectoparasites of bats. In this study, the complete mitochondrial genome of *P. jenynsii* was sequenced for the first time and a comprehensive phylogenetic analysis of the superfamily Hippoboscoidea was conducted. The complete mitochondrial genome of *P. jenynsii* is 16 165 base pairs (bp) in size, including 13 protein-coding genes (PCGs), 22 transfer RNA genes, 2 ribosomal RNA genes and 1 control region. The phylogenetic analysis based on 13 PCGs of the superfamily Hippoboscoidea known from the NCBI supported the monophyly of the family Nycteribiidae, and the family Nycteribiidae was a sister group with the family Streblidae. This study not only provided molecular data for the identification of *P. jenynsii*, but also provided a reference for the phylogenetic analysis of the superfamily Hippoboscoidea.

## Graphical abstract

**Figure:**
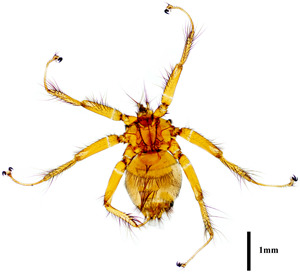

## Introduction

Nycteribiidae, belonging to the Dipteran superfamily Hippoboscoidea, is a specialized parasitic insect on the body surface of bats (Szentivanyi *et al*., 2016). More than 270 species of Nycteribiidae are known to exist (Dick and Graciolli, 2018). Together with the family Streblidae, they are called bat flies which feed on the blood of bats. As a member of the superfamily Hippoboscoidea, the Nycteribiidae also had a unique life history process that involves reproducing *via* viviparous puparity. Eggs are fertilized internally and all larval stages develop within the female, nourished by intrauterine “milk” glands. The larvae moult twice within the female and gravid females deposit a single terminal (third instar) larva on the roost substrate. Once deposited, the larva (called a prepupa) immediately forms a puparium. After a pupal stage of 3–4 weeks, an adult emerges and must find and colonize a host (Dick and Patterson, 2006). In recent years, bats and their surface parasites have received a great deal of attention as bat pathogens, such as 2019 novel coronavirus (Fan *et al*., 2019), have become widespread worldwide. Numerous studies have found zoonotic pathogens in bat flies, e.g. Bartonella (Low *et al*., 2022), narnaviruses, reoviruses (Xu *et al*., 2022). Therefore, it is hypothesized that bat flies are potential vectors for pathogen transmission. The monitoring of Nycteribiidae relies heavily on basic research on its biology, genetics, genomics, etc.

The mitochondrial genome is the only extra-nuclear genetic information carrier in animals, and it has the characteristics of lack of extensive recombination, conserved gene content, matrilineal inheritance, small molecular weight (approximately 14–21 kb), high mutation rate and a fast evolutionary rate (Yang *et al*., 2022). Therefore, mitochondrial DNA is often considered a useful molecular marker for species identification, phylogeography, analysis of population structure and dynamics and molecular evolution. Currently, only 4 species of Nycteribiidae in the Genebank database have undergone complete mitochondrial genome sequencing, which is grossly inconsistent with the rich species diversity of Nycteribiidae. Therefore, in order to understand the phylogenetic relationships of the superfamily Hippoboscoidea and further study the population genetics of Nycteribiidae, we sequenced the complete mitochondrial genome of *Penicillidia jenynsii* and analysed its characteristics and evolutionary relationships among the superfamily Hippoboscoidea.

## Materials and methods

### Sample collection and identification of morphological characteristics

Bat flies were collected from the body surface of captured *Miniopterus fuliginosus* (Chiroptera: Miniopterus) in July 2022 in Binchuan (100.58'E, 25.83'N), Yunnan Province, China, placed in sample tubes containing 95% ethanol and stored in a −20°C refrigerator until sample processing. In the laboratory, the collected bat flies were placed directly under the SZ2-ILST dissection microscope (Olympus, Tokyo, Japan) for species identification. One sample was mounted onto glass slides with Hoyer's solution. After dehydration, drying and transparency, the mounted specimen was photographed under a Leica DM 3000 LED microscope (Lecia, Weztlar, Germany) for pictures of the morphological features of the sample.

### DNA extraction, library preparation and mitogenome sequencing

The total DNA was extracted from the insects' whole body tissue using the Tissue DNA Kit (Omega Georgia, Connecticut, USA) according to the manufacturer's instructions. The DNA content was quantified using a NanoDrop One Microvolume UV-Vis Spectrophotometer (Thermo Scientific, Massachusetts, USA). A measure of 0.2 *μ*g DNA was used as the input material for DNA library preparation. The sequencing library was prepared using the NEBNext UltraTM DNA Library Prep Kit (New England Biolabs, New York, USA) for Illumina according to the manufacturer's recommendations, and index codes were added. DNA fragments were then end-polished, A-tailed and ligated with the full-length adapter for Illumina sequencing, followed by further polymerase chain reaction (PCR) amplification. After PCR, the products were purified using the AMPure XP system (Beverly, Los Angeles, USA). The quality of the libraries was then assessed using the Agilent 5400 system (Agilent, Palo Alto, USA) and quantified by quantitative PCR (1.5 nm). The qualified libraries were pooled and sequenced on Illumina platforms with the PE150 strategy at Novogene Bioinformatics Technology Co., Ltd. (Beijing, China) according to the effective library concentration and the required amount of data.

### Assembly, annotation and sequence analysis

This project used MitoZ 2.3 (https://doi.org/10.1101/489955) to assemble the mitochondrial genome. The 13 protein-coding genes (PCGs) and 2 ribosomal RNA genes (rRNA) of mitochondrial genome were annotated by MITOS (Donath *et al*., 2019), and manually compared with the known Nycteribiidae mitochondrial sequences. The 22 transfer RNA genes (tRNA) of the genome were annotated by tRNAscan-SE (Chan *et al*., 2021) and second structures of tRNAs were predicted by MITOS. Finally, the circular mitochondrial genome map was drawn using GENOMEVX (Conant and Wolfe, 2008). Codon W 1.4.2 (https://sourceforge.net/projects/codonw/) was used to calculate the composition of the base and the relative synonymous codon usage (RSCU). To calculate GC- and AT- skews, the following formulas were used: AT-skew = (A – T)/(A + T) and GC-skew = (G – C)/(G + C) (Perna and Kocher, 1995). MEGA 11 was used to estimate the genetic distances using the Kimura-2 parameter (K2P) (Tamura *et al*., 2021). The non-synonymous (Ka) to synonymous rate (Ks) ratio test of 13 PCGs was conducted in DnaSP 6 (Rozas *et al*., 2017).

### Phylogenetic analysis

The phylogenetic relationships of *P. jenynsii* with other species of the superfamily Hippoboscoidea, which could be found in the NCBI (Table 1), were constructed on the basis of 13 PCGs, selecting *Carcinoscorpius rotundicauda* and *Limulus polyphemus* as outgroups. Since tRNA genes are highly conserved, they were not considered when constructing the phylogenetic tree (Gray *et al*., 1984). Thirteen PCGs from *P. jenynsii* and other species available in GenBank were aligned using MAFFT 7, using default parameters (Katoh and Standley, 2013). Gblocks 0.91b software was used to remove the intergenic gaps and ambiguous sites (Castresana, 2000). The best-fit model was selected using PartitionFinder 2 (Lanfear *et al*., 2017). The PhyloSuite 1.2.2 was used to perform phylogenetic analyses using maximum likelihood (ML) and Bayesian inference (BI) methods (Zhang *et al*., 2020). For the ML analysis, an ML phylogenetic tree was constructed using IQ-TREE 2.1.2 with 1000 ultra-fast bootstrapping under the GTR + F + I + G4 model (Minh *et al*., 2020). The construction of Bayesian phylogenetic trees was performed using the MRBayes 3.2 software based on the best-fit partitioning scheme and corresponding models in Table S1 (Ronquist *et al*., 2012), in which the initial 25% of the sampled data were discarded as aged data. When the mean standard deviation of the split frequencies was below 0.01, we considered that smoothness was achieved and stopped the run. The phylogenetic tree was edited and visualized in the software FigTree.v1.4.4 (http://tree.bio.ed.ac.uk/software/figtree/).

**Table 1.** Mitochondrial genome information used in this study

Family	Species	Length (bp)	GenBank accession No.	References
Glossinidae	*Glossina austeni*	17 449	MZ826152	Porter *et al*. (2022)
Glossinidae	*Glossina brevipalpis*	17 751	MZ826153	Porter *et al*. (2022)
Hippoboscidae	*Melophagus ovinus*	15 573	KX870852	Liu *et al*. (2017)
Hippoboscidae	*Lipoptena* sp.	16 953	MT679542	Wang *et al*. (2021)
Hippoboscidae	*Ornithomya biloba*	18 654	MZ379837	Li *et al*. (2022)
Streblidae	*Paradyschiria parvula*	14 588	MK896865	Trevisan *et al*. (2019)
Streblidae	*Paratrichobius longicrus*	16 296	MK896866	Trevisan *et al*. (2019)
Nycteribiidae	*Basilia ansifera*	16 964	MZ826150	Porter *et al*. (2022)
Nycteribiidae	*Dipseliopoda setosa*	19 164	MZ826151	Porter *et al*. (2022)
Nycteribiidae	*Nycteribia parvula*	16 060	OP442519	Unpublished
Nycteribiidae	*Phthiridium szechuanum*	14 896	OP459298	Unpublished
Limulidae	*Carcinoscorpius rotundicauda*	15 037	JX437074	Baek *et al*. (2014)
Limulidae	*Limulus polyphemus*	14 985	NC_003057	Lavrov *et al*. (2000)

## Results

### Morphological features of *P. jenynsii*

**Figure 1.**
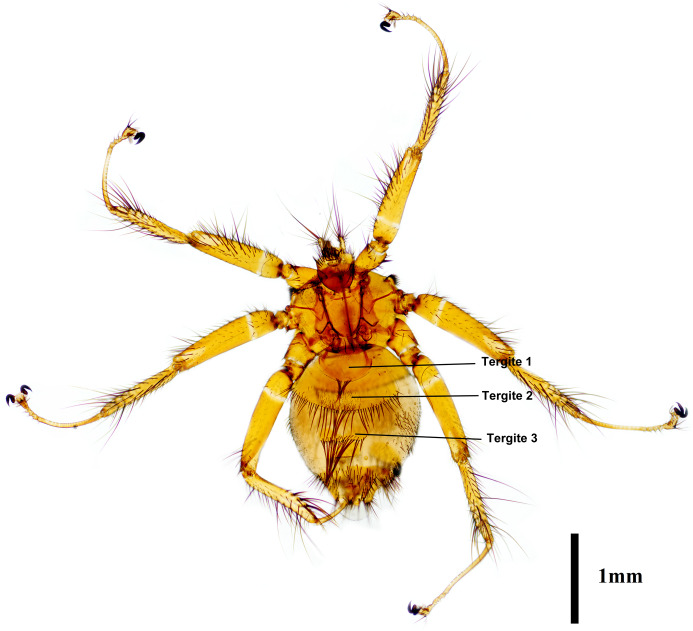
Dorsal side of female *Penicillidia jenynsii*.

The female abdomen was the main point of identification for *P. jenynsii* (Fig. 1). Tergite 1 was rounded, with 2 groups of 2–4 moderately long setae in the middle of the posterior margin. Tergite 2 had a marginal row of long setae in the middle and shorter setae laterally, as well as a group of short setae on the surface that may or may not have been present. Tergite 3 was broad and bare on the surface, with a marginal row of long and short setae. The anal segment was conical, with short hairs on the dorsal surface near the base. The connexivum between the tergal plates was bare (Rothschild and Theodor, 1967).

### Mitochondrial genome structure and base composition

**Figure 2.**
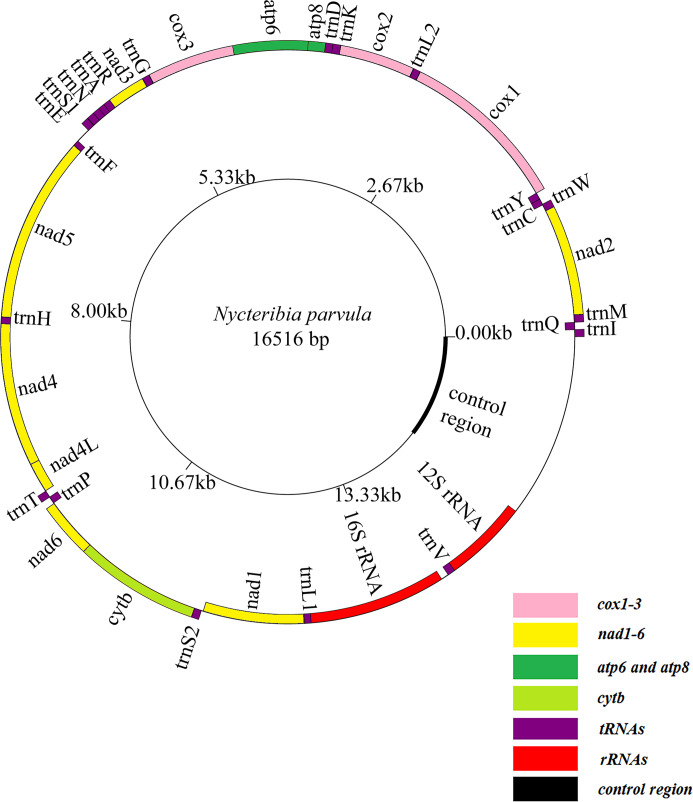
The circular mitochondrial genome map of *Penicillidia jenynsii*.

The complete mitochondrial genome of *P. jenynsii* is a circular, double-stranded structure of 16 516 bp in length, consisting of 13 PCGs, 22 tRNAs, 2 rRNAs and the control region (Table 2). The A, T, G and C base compositions were 40.26%, 41.88%, 6.64% and 11.21%%, respectively. The AT content was 82.15%, with a strong AT preference. The AT-skew was −0.02, and the GC-skew was −0.26. In the complete mitochondrial genome, 23 genes are located in the majority strand (J-strand), including 9 PCGs and 14 tRNAs. The other genes are located on the minority strand (N-strand), including 4 PCGs, 8 tRNAs and 2 rRNAs (Fig. 2). The accession number for GeneBank is OQ127278.

**Table 2.** Mitochondrial genome organization of *Penicillidia jenynsii*

Gene	Coding strand	Position and length (bp)	Nucleic acid		Codon		AT-skew	GC-skew	Anticodon	Intergenic nucleotides
			AT%	GC%	Start	Stop				
*trnI*	J	1–67 (67)	80.60	19.40					GAT	−2
*trnQ*	N	65–133 (69)	82.61	17.39					TTG	−1
*trnM*	J	133–200 (68)	75.00	25.00					CAT	0
*nad2*	J	201–1196 (996)	85.04	14.96	ATT	TAA	−0.18	−0.23		−2
*trnW*	J	1195–1262 (68)	73.53	26.47					TCA	−8
*trnC*	N	1255–1314 (60)	68.33	31.67					GCA	0
*trnY*	N	1315–1378 (64)	76.56	23.44					GTA	−2
*cox1*	J	1377–2910 (1534)	73.60	26.40	TCG	T	−0.15	−0.09		1
*trnL2*	J	2912–2974 (63)	77.78	22.22					TAA	2
*cox2*	J	2977–3655 (679)	77.32	22.68	ATG	T	−0.09	−0.14		0
*trnK*	J	3656–3726 (71)	70.42	29.58					CTT	−1
*trnD*	J	3726–3791 (66)	89.39	10.61					GTC	0
*atp8*	J	3792–3953 (162)	88.89	11.11	ATT	TAA	0.03	−0.56		−7
*atp6*	J	3947–4621 (675)	79.11	20.89	ATG	TAA	−0.19	−0.34		−1
*cox3*	J	4621–5409 (789)	78.71	21.29	ATG	TAA	−0.12	−0.13		2
*trnG*	J	5412–5476 (65)	89.23	10.77					TCC	0
*nad3*	J	5477–5827 (351)	82.91	17.09	ATT	TAG	−0.18	−0.13		−2
*trnA*	J	5826–5888 (63)	73.02	26.98					TGC	−1
*trnR*	J	5888–5948 (61)	77.05	22.95					TCG	−3
*trnN*	J	5946–6009 (64)	75.00	25.00					GTT	0
*trnS1*	J	6010–6077 (68)	76.47	23.53					GCT	3
*trnE*	J	6081–6147 (67)	94.03	5.97					TTC	136
*trnF*	N	6284–6337 (54)	85.19	14.81					GAA	0
*nad5*	N	6338–8075 (1738)	83.43	16.57	TTG	T	−0.12	−0.27		0
*trnH*	N	8076–8139 (64)	82.81	17.19					GTG	0
*nad4*	N	8137–9469 (1330)	82.41	17.59	ATG	T	−0.18	−0.37		−1
*nad4L*	N	9469–9747 (279)	85.30	14.70	ATG	TAA	0.15	−0.66		2
*trnT*	J	9750–9815 (66)	86.36	13.64					TGT	0
*trnP*	N	9815–9880 (65)	87.69	12.31					TGG	5
*nad6*	J	9886–10 389 (504)	88.10	11.90	ATT	TAA	−0.14	−0.47		−1
*Cytb*	J	10 389–11 516 (1128)	78.10	21.90	ATG	TAA	−0.11	−0.27		1
*trnS2*	J	11 518–11 584 (67)	82.09	17.91					TGA	16
*nad1*	N	11 601–12 536 (936)	79.49	20.51	ATG	TAG	0.18	−0.32		4
*trnL1*	N	12 541–12 601 (61)	80.33	19.67					TAG	0
*16S rRNA*	N	12 602–13 874 (1273)	83.82	16.18	TAA	T				71
*trnV*	N	13 946–14 016 (71)	81.69	18.31					TAC	0
*12S rRNA*	N	14 017–14 799 (783)	81.10	18.99	ATA	ATG				0

### Description of PCGs

There are 13 PCGs (Fig. 2). Except for 2 PCGs that start with TCG (*cox1*) and TTG (*nad5*), the other 11 PCGs start with standard ATN codons (4 ATT and 7 ATG). Except for 4 PCGs (*cox1*, *cox2*, *nad4* and *nad5*) that ended with incomplete stop codons T, the remaining 11 PCGs (7 TAA and 2 TAG) have complete stop codons.

**Figure 3.**
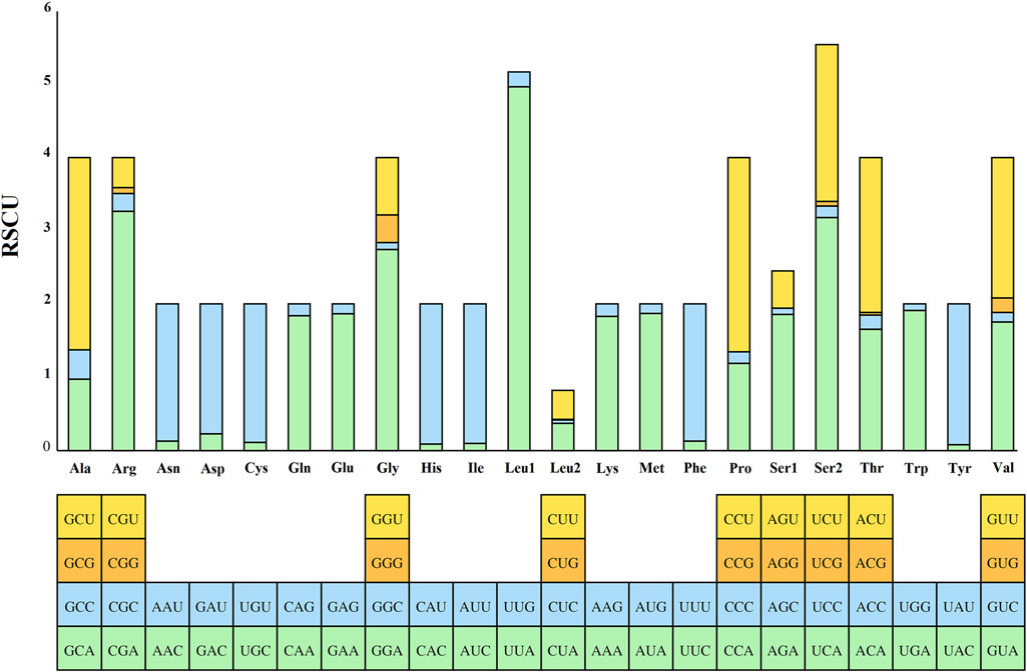
Relative synonymous codon usage (RSCU) of *Penicillidia jenynsii.*

PCGs' codon usage and RSCU were calculated (Fig. 3). The results show that UUA, AUU, UUU and AUA were the most frequently used codons among the *P. jenynsii*'s mitochondrial PCGs, and GCG, CCG and AGG were less frequently used codons.

**Figure 4.**
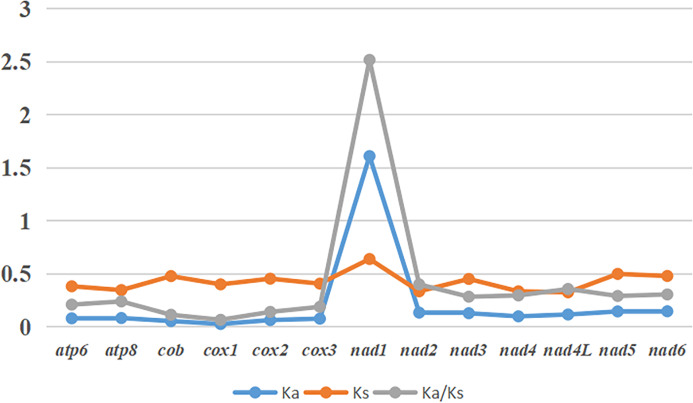
Ka, Ks and Ka/Ks of 13 PCGs of *Penicillidia jenynsii.*

The Ka, Ks and Ka/Ks were computed for each PCG, and details are shown in Fig. 4. The values of Ka/Ks ranged from 0.07 to 2.52. The evolution rate of 13 PCGs was sequentially *nad1* > *nad2* > *nad4L* > *nd6* > *nad4* > *nad5* > *nad3* > *atp8* > *atp6* > *cox3* > *cox2* > *cytb* > *cox1.* Among them, *nad1* was positively selected (Ka/Ks > 1), while other genes were purified (Ka/Ks < 1).

### tRNA, rRNA genes and control region

The length of the 22 tRNAs ranges from 54 to 71 bp. All tRNAs fold into a typical cloverleaf structure except *trnF* that lacked the TΨC arms and *trnS1* that lacked the dihydrouracil (DHU) arm (Fig. S1). The *16S rRNA* was 1273 bp in length with 83.82% AT content, which was located between *trnL1* and *trnV*. The *12S rRNA* was 789 bp in length with 81.10% AT content, which was located between *trnV* and the control region. The control region was located between *trnV* and *trnI*, with a sequence length of 1717 bp. Furthermore, the AT content was 90.62%.

### Phylogenetic analysis of *P. jenynsii*

#### Genetic distance

The analysis of corrected genetic distances was performed to investigate the phylogenetic relationships of the superfamily Hippoboscoidea (Table 3). The range of genetic distance is from 0.114 to 0.298. The average genetic distance is 0.270. Among the genetic distances of *P. jenynsii* and others, the genetic distance between *P. jenynsii* and *Phthiridium szechuanum* is the smallest (0.138).

**Table 3.** Estimation of pairwise genetic distances (%) among the species of the superfamily Hippoboscoidea

	1	2	3	4	5	6	7	8	9	10	11
1. *Penicillidia jenynsii*											
2. *Basilia ansifera*	0.175										
3. *Dipseliopoda setosa*	0.211	0.219									
4. *Glossina austeni*	0.294	0.298	0.295								
5. *Glossina brevipalpis*	0.273	0.275	0.274	0.177							
6. *Lipoptena* sp	0.268	0.270	0.267	0.224	0.208						
7. *Melophagus ovinus*	0.274	0.269	0.272	0.236	0.216	0.138					
8. *Nycteribia parvula*	0.141	0.172	0.210	0.296	0.274	0.263	0.266				
9. *Ornithomya biloba*	0.282	0.286	0.285	0.209	0.200	0.189	0.202	0.283			
10. *Paradyschiria parvula*	0.287	0.281	0.282	0.273	0.260	0.260	0.263	0.280	0.270		
11. *Paratrichobius longicrus*	0.268	0.274	0.266	0.255	0.241	0.233	0.238	0.264	0.243	0.204	
12. *Phthiridium szechuanum*	0.138	0.168	0.207	0.292	0.269	0.268	0.270	0.114	0.282	0.281	0.262

#### Phylogenetic analysis

**Figure 5.**
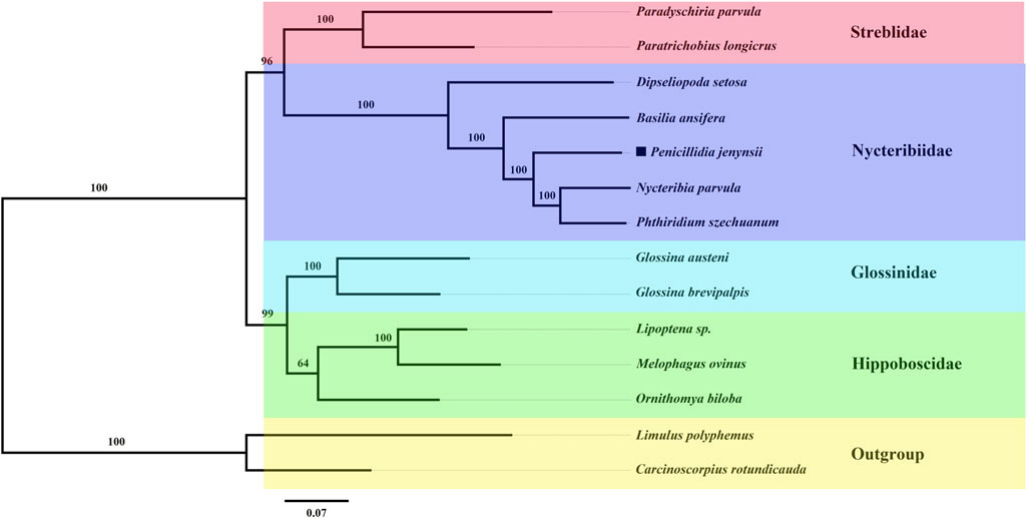
Phylogenetic relationships of the superfamily Hippoboscoidea using ML analyses based on 13 PCGs of mitogenomes.

**Figure 6.**
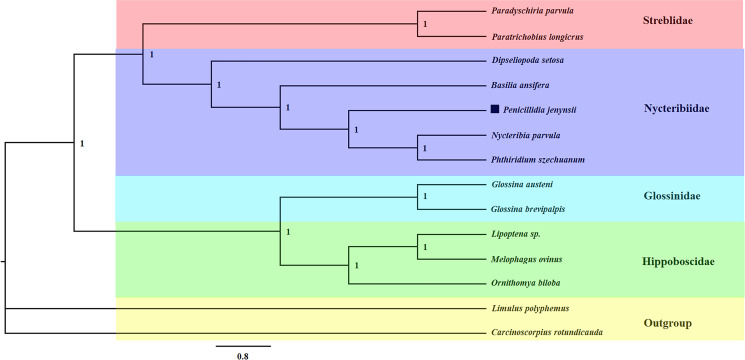
Phylogenetic relationships of the superfamily Hippoboscoidea using BI analyses based on 13 PCGs of mitogenomes.

The topologies of the phylogenetic tree completed by the 2 methods were identical, with high support for each node (Figs 5 and 6). The results demonstrate the superfamily Hippoboscoidea is divided into 2 branches: one is Nycteribiida and Streblidae, and the other is Glossinidae and Hippoboscidae. The family Nycteribiidae is a monophyletic and sister group with the family Streblidae. This is consistent with the morphological classification. *Penicillidia jenynsii* has the closest affinity to *P. szechuanum* and *Nycteribia parvula*.

## Discussion

In this study, we sequenced the complete mitochondrial genome of *P. jenynsii* for the first time and conducted a comprehensive phylogenetic analysis based on 13 PCGs of *P. jenynsii* and the other 11 species. The sequence of the mitochondrial genome of *P. jenynsii* is the same as that known in the superfamily Hippoboscoidea and is identical to the sequence of *Drosophila melanogaster* which is the classical structure of Diptera (Lewis *et al*., 1995). Mitochondrial genome rearrangements as evolutionary events are relatively rare; therefore, this is an important tool for assessing phylogenetic relationships between species. Eight of the 13 PCGs have the classical ATN as the start codon, while *cox1* has TCG as the start codon, which is common in species of the superfamily Hippoboscoidea (Tang *et al*., 2018; Porter *et al*., 2022). Nine of the 13 PCGs end with complete stop codons, while 4 genes (*cox1*, *cox2*, *nad4*, *nad5*) utilize incomplete stop codon T, which could be completed as TAA *via* posttranscriptional polyadenylation (Ojala *et al*., 1981). The tRNA can form a cloverleaf secondary structure except for the absence of the DHU arm of *trnS1* and the absence of the TΨC arms of *trnF*. The absence of the DHU arm of *trnS1* is a common phenomenon in Metazoa, and tRNA genes with incomplete secondary structures can maintain their normal functions through post-transcriptional modifications (Negrisolo *et al*., 2011). The positions of these 2 rRNAs are more conserved as in the other Dipteran mitochondrial genomes. The control region, also known as the AT-enriched region, has an AT content of 90.62% in *P. jenynsii* and is thought to control the replication and transcription of the mitochondrial genome (Boore, 1767). The length variation among insect mitochondrial genomes depends mainly on the variation in the A + T-rich region, which ranges from 70 to 13 kb in length (Zhang and Hewitt, 1997).

The base composition of the mitochondrial genome shows a severe bias, with 82.15% AT content, similar to other species in the superfamily Hippoboscoidea and it has been suggested that the species with higher AT content in mitochondrial genes are considered to be more evolutionarily advantageous (Vrijenhoek, 1994). The results of AT-skew and GC-skew could reflect the preference for A and G bases in the mitochondrial genome (Lindahl, 1993). The main causes of base skewness are asymmetric mutation of bases during replication and transcription, as well as selection pressure.

Ka/Ks values of PCGs can reflect the selective pressure on the gene and, to some extent, the conservativeness of the gene; Ka/Ks < 1 is considered purifying selection; Ka/Ks > 1 is considered positive selection. The smaller the value of Ka/Ks, the greater the selective pressure on the gene, and the more conservative the gene; the larger the value of Ka/Ks, the faster the evolutionary rate of the gene, and the less conservative the gene (Nekrutenko *et al*., 2002). In the mitochondrial genome, the Ka/Ks values of all PCGs except *nad1* are less than 1. It is evident that most of the PCGs are conserved, among which the Ka/Ks values of *cox1* and *cytb* are significantly smaller than those of other genes, indicating that they are under strong selection pressure and the genes are more conserved. On the contrary, the Ka/Ks values of *nad1* and *nad2* were relatively larger, and these genes were under weaker selection pressure and were relatively less conservative. Other studies have also shown that the *cox1* and *cytb* genes are the most conserved in the mitochondrial genome, which are now widely used for molecular markers and phylogenetic studies (Park *et al*., 2010; Tobe *et al*., 2010).

Two methods of completing the phylogenetic trees of the superfamily Hippoboscoidea revealed that the families Nycteribiidae, Hippoboscidae, Glossinidae and Streblidae are monophyletic, and the species within these families clustered together with high confidence. The families Hippoboscidae, Nycteribiidae, Streblidae and Glossinidae have been combined into 1 superfamily because of their unique reproductive mode, adenotrophic viviparity (Petersen *et al*., 2007). Except for family Glossinidae which is free-living, the other 3 families are all true ectoparasites, spending all or most of their adult lives within the fur or feathers of their mammalian and bird hosts, and thus undergo parasitic adaptations (McAlpine, 1989). This, together with the relatively small amount of molecular data on Hippoboscoidea species, has led to disagreement in the classification of Hippoboscoidea. Initially, only 3 families of species, Hippoboscidae, Nycteribiidae and Streblidae, were considered to belong to the superfamily Hippoboscoidea, and some experts would classify Nycteribiidae and Streblidae into Hippoboscidae (Petersen *et al*., 2007). In this study, all PCGs of the mitochondrial genes were analysed phylogenetically to provide definitive evidence for the classification of the superfamily Hippoboscoidea.

## Data Availability

The genome sequence data that support the findings of this study are openly available in GenBank of NCBI at https://www.ncbi.nlm.nih.gov/, reference number OQ127278. The associated SRA number is SRR23882031.

## Supplementary material

For supplementary material accompanying this paper visit https://doi.org/10.1017/S003118202300032X. click here to view supplementary material
