# Supplementary material for: Complete mitochondrial genome of Penicillidia jenynsii (Diptera: Hippoboscoidea: Nycteribiidae) and phylogenetic relationship
Source: Parasitology. 2023 Mar 27;150(7):623–30. doi: 10.1017/S003118202300032X (PMC10260298; doi:10.1017/S003118202300032X)
Supplement: Supplementary file 1 [file S003118202300032Xsup001.docx]

**Table S1.** The best-fit partitioning scheme and corresponding models used on the BL phylogenetic tree.

| Subset partitions | Sites | Best model |
| --- | --- | --- |
| *nad3* codon1, *atp6* codon1, *nad6* codon1, *nad2* codon1, *atp8* codon2, *atp8* codon1 | 972 | GTR+I+G |
| *cox1* codon2, *cox2* codon2, *cytb* codon2, *cox3* codon2, *atp6* codon2, *nad3* codon2 | 1698 | TVM+I+G |
| *cox1* codon3, *cox3* codon3, *cox2* codon3, *nad3* codon3, *cytb* codon3, *atp6* codon3 | 1698 | TRN+G |
| *nad6* codon3, *nad2* codon3, *atp8* codon3 | 575 | TRN+G |
| *cox1* codon1, *cox2* codon1, *cytb* codon1, *cox3* codon1 | 1353 | GTR+I+G |
| *nad4L* codon1, *nad4* codon1, *nad1* codon1, *nad5* codon1 | 1462 | GTR+I+G |
| *nad4L* codon2, *nad5* codon2, *nad4* codon2, *nad1* codon2 | 1462 | GTR+G |
| *nad1* codon3, *nad5* codon3, *nad4L* codon3, *nad4* codon3 | 1462 | TRN+I+G |
| *nad2* codon2, *nad6* codon2 | 523 | TVM+G |


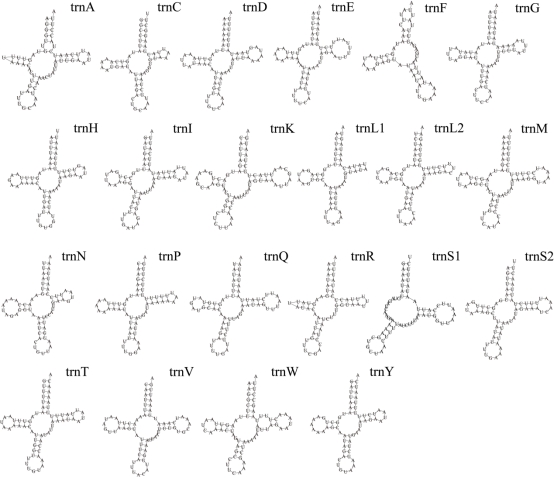


**Figure S1** The secondary structure of 22 tRNA in *Penicillidia jenynsii.*
